# Great Trees

**Published:** 1898-09

**Authors:** 


Great Trees. In Gypsyland, Australia, the eucalyptus tree, species *E. globulus*, often attains a height of 450 feet, and is considered by botanists to be the tallest tree on the globe. Pure oil of eucalyptus is principally obtained from the leaves of this species.

